# An Efficient LiDAR Point Cloud Map Coding Scheme Based on Segmentation and Frame-Inserting Network

**DOI:** 10.3390/s22145108

**Published:** 2022-07-07

**Authors:** Qiang Wang, Liuyang Jiang, Xuebin Sun, Jingbo Zhao, Zhaopeng Deng, Shizhong Yang

**Affiliations:** 1 College of Information and Control Engineering, Qingdao University of Technology, Qingdao 266525, China; wangqiang@qut.edu.cn (Q.W.); jiangliuyang@qut.edu.cn (L.J.); zhaojbqut@163.com (J.Z.); dengzhaopeng@qut.edu.cn (Z.D.); 2 State Key Laboratory of Precision Measuring Technology and Instruments, Tianjin University, Tianjin 300072, China; 3 School of Automation Science and Electrical Engineering, Beihang University, Beijing 100083, China; 4 School of Electronics and Information Engineering, Shenzhen University, Shenzhen 518060, China; sunxuebin@szu.edu.cn

**Keywords:** LiDAR, point cloud map, coding, segmentation, interpolation

## Abstract

In this article, we present an efficient coding scheme for LiDAR point cloud maps. As a point cloud map consists of numerous single scans spliced together, by recording the time stamp and quaternion matrix of each scan during map building, we cast the point cloud map compression into the point cloud sequence compression problem. The coding architecture includes two techniques: intra-coding and inter-coding. For intra-frames, a segmentation-based intra-prediction technique is developed. For inter-frames, an interpolation-based inter-frame coding network is explored to remove temporal redundancy by generating virtual point clouds based on the decoded frames. We only need to code the difference between the original LiDAR data and the intra/inter-predicted point cloud data. The point cloud map can be reconstructed according to the decoded point cloud sequence and quaternion matrices. Experiments on the KITTI dataset show that the proposed coding scheme can largely eliminate the temporal and spatial redundancies. The point cloud map can be encoded to 1/24 of its original size with 2 mm-level precision. Our algorithm also obtains better coding performance compared with the octree and Google Draco algorithms.

## 1. Introduction

LiDAR point clouds have been widely used in many emerging applications [1], such as the preservation of historical relics, mobile robots, and remote sensing [2,3,4,5,6,7,8,9]. LiDAR sensors are essential for autonomous vehicles, and dense LiDAR point cloud maps play an indispensable role in unmanned driving, such as obstacle detection [10], localization [11], and navigation [12]. In wide geographic areas, a LiDAR point cloud map consists of a vast number of points and requires a large bandwidth and storage space to transmit and store [4,13,14,15]. Therefore, developing compression algorithms for the dense LiDAR point cloud maps is an urgent task.

With the characteristics of covering a large area, unstructured organization, and a huge volume [16], it is difficult to remove redundancy without distortion when encoding the LiDAR point cloud map. Octree, as a data structure, has been widely used to encode point clouds. Each internal node has exactly eight children in an octree. The octree-based point cloud compression method is to divide a 3D point cloud by recursively subdividing it into eight octants. As octree-based compression methods are lossy, they are suboptimal for the autonomous vehicles that have strict requirements for compression accuracy in the task of path planning or obstacle detection, etc.

In this research, we focus on compressing the large-scale dense LiDAR point cloud maps. The well-known low-drift and real-time LiDAR odometry (LOAM) algorithm are used to build the 3D map [17]. The proposed LiDAR point cloud map coding algorithm can be used in mobile robots or surveying and mapping fields. The major contributions are as follows.

- By recording the time stamp and quaternion matrix of each scan during mapping, the large-scale point cloud map compression can be formulated as a point cloud sequence compression problem;
- For intra-coding, we develop an intra-prediction method based on segmentation and plane fitting, which can exploit and remove the spatial redundancy by utilizing the spatial structure characteristics of the point cloud.
- For inter-coding, we develop an interpolation-based inter-prediction network, in which the previous time and the next time encoded point clouds are utilized to synthesize the point clouds of the intermediate time to remove the temporal redundancy.
- Experimental results on the KITTI dataset demonstrate that the proposed method achieves a competitive compression performance for the dense LiDAR point cloud maps compared with other state-of-the-arts.

## 2. Point Cloud Coding: A Brief Review

The compression of the 3D point cloud data is a hot topic and preliminary investigations have been recently made.

### 2.1. Volumetric/Tree-Based Point Clouds Coding

For unstructured point clouds, Octree representation is commonly utilized as a point-cloud geometry compression method. To improve the immersive visual experience, De Oliveira Renteet et al. [18] proposed an efficient geometric coding scheme for point clouds, in which an octree-based compression algorithm compression was utilized as a basic layer and used the graph transformation technique as an enhancement layer to encode the residual data. Their reported evaluation results show that the method produced significant improvement, especially at low and medium bit rates. Traditional point-cloud compression algorithms are limited to encoding the position and attribute of discrete point clouds. In [19], Krivokua et al. introduced an alternative technique based on volume function. Like regression analysis, the volume function is a continuous function that can interpolate the values on a finite set of points into a linear combination of continuous basis functions. B-spline wavelet basis is utilized for encoding the volume function, which represents the geometry and attributes of point clouds. Compared to the latest MPEG point-cloud coding standard [20], their algorithm achieves better coding performance in geometry and attributes. To alleviate the computational complexity of the 3D point-cloud model in registration, data abstraction, and visualization, Elseberg et al. [21] proposed an effective point-cloud storage and compression scheme based on the Octree. The coding scheme can be used in file format conversion and 3D model registration.

### 2.2. Image/Video-Based Point Clouds Coding

For structured point clouds, some studies have focused on employing image/video codecs to compress point cloud data by mapping it into 2D data. Similar to this approach, Tu et al. [22] transformed LiDAR point clouds into a range image sequence and used a simultaneous localization and mapping (SLAM) algorithm to perform inter-prediction. The intra-frame and inter-prediction residual data are encoded by the MPEG-like compression method. Unlike the aforementioned methods, Wang et al. [23] proposed a method to compress RGB-D point clouds. The properties between RGB-D point clouds and LiDAR point clouds are similar, except for the measurement range (i.e., Lidar is around 100 m, while RGB-D camera is around a few meters). They developed a warping-based depth data coding method, in which a point-cloud registration algorithm was utilized to remove redundancy. Experimental results showed that their algorithm achieved a higher compress ratio with less distortion compared to recent methods. Tu et al. [24] used the conventional image and video-based schemes to compress the 2D arrays by converting the LiDAR data to a range image. Feng et al. [25] proposed a real-time spatio-temporal LiDAR point clouds compression scheme. In this scheme, key frames are identified and encoded by interative plane fitting, and then the temporal streams are encoded by referencing the spatially encoded data. In [26], Tu et al. firstly chose frames as keyframes (I-frame) and obtained the optical flow between the two nearest keyframes. Then, according to the two keyframes and the optical flow, a U-net network was utilized to generate the remaining LiDAR frames (P-frames) between the two keyframes. They removed the temporal redundancy by interpolating point cloud data between two non-adjacent frames. In [27], Tu et al. proposed an RNN-based network to encode LiDAR point clouds. They used a recurrent neural network, and only the input of the first layer was the point cloud data, while the inputs of other layers were the residual data. Their method was to remove the spatial redundancy of a frame of point clouds, not the temporal redundancy. Coding each frame is independent and does not depend on other frames.

### 2.3. Summary

Currently, there is still no efficient coding solution for the LiDAR point cloud map. Though volumetric/tree-based schemes, such as Google Draco [28] and MPEG TMC 13 [20], can be used to encode the LiDAR point cloud map, their coding performance is far from satisfactory as these methods fail to utilize the spatial structure of point clouds. Converting LiDAR data into a 2D matrix in itself is an efficient method for reducing spatial redundancy. However, most of the existing methods [22,24,29] directly use the image/video-based method to encode LiDAR range image without further exploiting the temporal and spatial redundancies.

## 3. Overall Codec Architecture

In the paper, the problem of efficient coding dense LiDAR point cloud map is addressed, which can be used for mobile robots or surveying and mapping fields. The low-drift and real-time LOAM algorithm is utilized to construct a LiDAR point cloud map [17]. During the mapping process, the time stamp of the LiDAR scans and quaternion matrices is recorded relative to the global coordinate origin. As the LiDAR point cloud map is constructed by these frames, the dense point cloud map compression can be specified into a point cloud sequence coding task.

**Figure 1:**
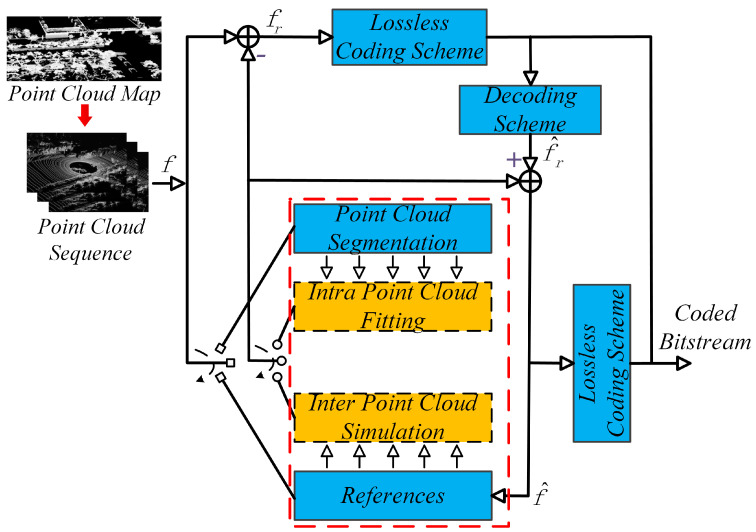
System architecture of our LiDAR point cloud map encoder.

Generally, the point cloud sequence compression needs to exploit both temporal and spatial redundancies. The system architecture of our LiDAR point cloud map coding algorithm is illustrated in Figure 1. We divide the frames in the point cloud sequence into intra-frames (I-frames) and inter-frames (B-frames, bi-prediction). An I-frame is compressed independently by removing the spatial redundancies, while a B-frame is compressed by referring to encoded I-frames or B-frames to remove the temporal redundancies. Two I-frames are encoded firstly, followed by B-frames in the middle of two I-frames. B-frames cannot be encoded independently and rely on two encoded I-frames [30].

The intra-frames are coded by a segmentation-based prediction technique, while for inter-frames, we develop an interpolation-based coding network to remove the temporal redundancy.

Decoding is the inverse process of encoding. The decoded residual data is added to the prediction data to recover the point clouds. According to the decoded point clouds, quaternion matrices, and translation matrices, the point cloud map can be reconstructed:

(1) $$\left[\begin{array}{c} x_{B}\\ y_{B}\\ z_{B} \end{array}\right]=R_{yaw}\times R_{pitch}\times R_{roll}\times \left[\begin{array}{c} x_{I}\\ y_{I}\\ z_{I} \end{array}\right]+\left[\begin{array}{c} C_{x}\\ C_{y}\\ C_{z} \end{array}\right],$$

where $x_{I}$, $y_{I}$, $z_{I}$ is *x*, *y*, *z* of coordinate system of an intra frame, and $x_{B}$, $y_{B}$, $z_{B}$ are corresponding *x*, *y*, *z*, when using the predicted B-frame as the coordinate origin. $C_{x}$, $C_{y}$, and $C_{z}$ denotes the translation matrix, and $R_{yaw}$, $R_{pitch}$, $R_{roll}$ represents the rotation matrix of yaw, pitch, and roll angle.

## 4. Intra-Frame Point Cloud Coding Based on Semantic Segmentation

### 4.1. Overview of Intra-Coding Network

**Figure 2:**
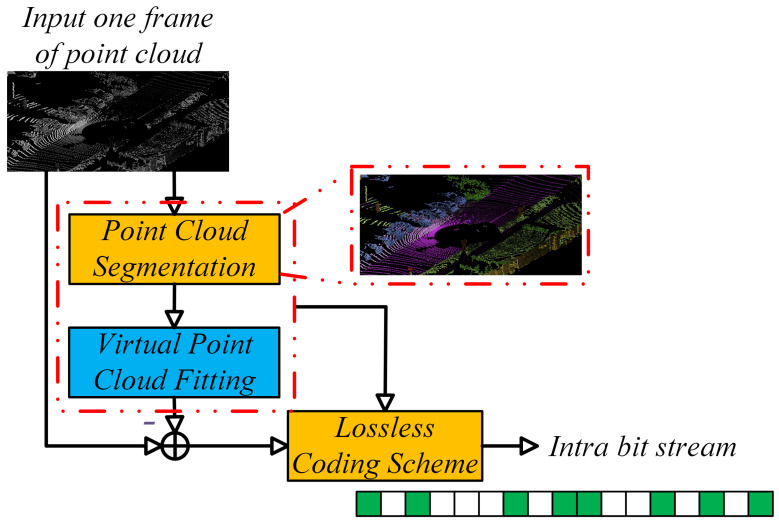
Intra-prediction technique based on segmentation.

The pipeline of the proposed segmentation-based intra-frame point cloud coding method is illustrated in Figure 2. Firstly, the point cloud conversion from 3D to 2D is performed to obtain the 2D Matrix of point clouds. Then, the RangeNet++ [31] network is utilized to realize point cloud segmentation. The residual data, contour map, and the quadric surface parameters are encoded with lossless coding schemes and packaged as the the intra-bitstream.

### 4.2. LiDAR Point Cloud Segmentation

The LiDAR data from the KITTI dataset is utilized to verify our method, which uses 64 channels, covering 26.9${}^{\circ }$ vertical field of view and a 360${}^{\circ }$ horizontal field of view [32]. Considering that LiDAR sensors acquire data in an orderly way, the point clouds can be converted from $R^{3}$ to $R^{2}$. Let ${\left(I_{i}=\left(x_{i},y_{i},z_{i}\right)\right)}_{i=1\ldots N}$ be the coordinates of a frame of point cloud captured by Velodyne sensors. Considering that the point clouds is ordered, it can be transformed into a 2D matrix $X{\left(u,v\right)}_{u=1\ldots M,v=1\ldots N}$.

(2) $$\left(\begin{array}{c} u\\ v \end{array}\right)=\left(\begin{array}{c} \frac{1}{2}\left(1-arctan\left(y,x\right)\pi^{-1}\right)\cdot w\\ (1-\left(arcsin\left(zr^{-1}+f_{up}\right)\right)f^{-1})\cdot h \end{array},\right)$$

where (x,y,z) represent the coordinates of point *P*, (u,v) are 2D matrix coordinates, (h,w) are the height and width of the desired 2D matrix representation, $r=\sqrt{x^{2}+y^{2}+z^{2}}$ represents the distance of point P=(x,y,z) from the origin, and $f=f_{up}+f_{down}$ denotes the vertical field of view of the LiDAR sensor [33].

The RangeNet++ takes the fused 2D information as input and outputs the segmentation results. Three 2D convolutional blocks are adopted as a 2D feature extractor. The output of the final layer is the point cloud segmentation results. The segmentation results will pave the way for the subsequent surface fitting-based intra-prediction technique.

### 4.3. Segmentation-Based Intra-Prediction Technique

Nearly one-third of the point cloud data are ground points. After segmenting the point cloud, we can segment the ground and other objects. Thus, the quadric surface fitting-based technique is utilized to fit the point clouds. Considering the complexity and compression efficiency, we fit the segmented region with the plane for the ground points and sphere for other object.

A plane is defined by J=(n,d), where *n* is the normal vector, with ||n||=1, and *d* is the vertical distance from the origin to the plane. The distance from a point $p_{i}$ to the plane is defined as:

(3) $$D_{plane}\left(J,p_{i}\right)=\left|\right|n^{T}p-d\left|\right|$$

Then we can construct the equation $\varepsilon_{plane}$ as a function of the distance of the minimum value of the sum.

(4) $$\varepsilon_{plane}\left(J,S\right)=\sum_{i=1}^{N}\left|\right|n^{T}S_{i,:}-d\left|\right|$$

A sphere is represented by J=(c,r), where r∈R denotes the radius, and $c\in R^{3}$ represents the center. The distance from point $p_{i}$ to the sphere is defined as:

(5) $$D_{sphere}\left(J,p_{i}\right)=||c-p_{i}\left|\right|-r$$

Then we can construct the fitting equation $\varepsilon_{sphere}$ as a function of the distance of the minimum value of the sum.

(6) $$\varepsilon_{sphere}\left(J,S\right)=\sum_{i=1}^{N}||(||c-p_{i}\left||\right)-r||$$

### 4.4. Residual Data Coding

According to the parameters of the LiDAR sensor, we use the fitting plane to calculate the coordinates of the virtual points. The residual data $R_{intra}\left(x,y\right)$ is the difference between the original range image $X_{intra}\left(x,y\right)$ and the predicted range image $P_{intra}\left(x,y\right)$. As the pixel values of the residual data are nearly zero, the entropy of the $R_{intra}\left(x,y\right)$ is smaller copared with $X_{intra}\left(x,y\right)$. Thus, the residual data $R_{intra}\left(x,y\right)$ can be encoded with fewer bits.

(7) $$R_{intra}\left(x,y\right)=X_{intra}\left(x,y\right)-P_{intra}\left(x,y\right).$$

## 5. Inter-Frame Point Cloud Coding Based on Inserting Network

### 5.1. Overall Inter-Prediction Network

**Figure 3:**
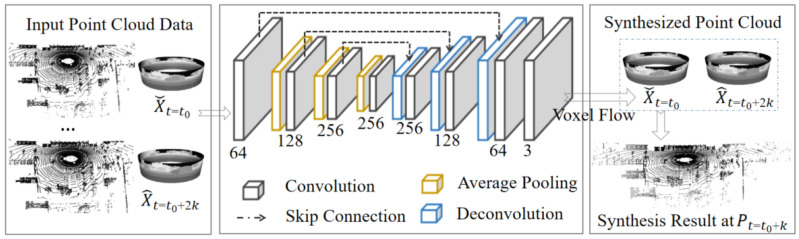
Inter-frame point cloud inserting network.

To remove temporal redundancy in the LiDAR point clouds [34], an inter-frame point cloud inserting network is designed, as illustrated in Figure 3. The interpolation module utilizes the encoded points clouds $X=\{X_{t0},X_{t0+2k}\}$, and generates the internal point cloud frame $P_{t0+k}$. We calculate the difference between the predicted result $P_{t0+k}$ and the real point cloud $X_{t0+k}$ as residual data $R_{inter}\left(x,y\right)$, which will be encoded as the inter-bitstream.

### 5.2. Point Cloud Interpolation Module

Figure 3 illustrates the diagram of the LiDAR point cloud interpolation module. The encoder and decoder parts predict the 3D voxel stream, which will be used to generate the required intermediate frames. The network generate the predicted frame $P_{t=t_{0}+k}$ according to the input point clouds ${\overset{ˇ}{X}}_{t=t_{0}}$ and ${\hat{X}}_{t=t_{0}+2k}$, where ${\overset{ˇ}{X}}_{t=t_{0}}$, ${\hat{X}}_{t=t_{0}+2k}$ are already encoded frames. The predicted frame can be the middle frame by interpolating or the next frame by extrapolating the input point clouds. We focus on interpolating the intermediate frame according to two decoded frames. The network is represented by $H(X_{rec},\Theta )$, where the output *F* is the 3D voxel flow of the input $X_{rec}$, and Θ is the network parameters.

(8) $$F=\left(\Delta x,\Delta y,\omega \right)=H\left(X_{rec};\Theta \right),$$

where *F* is the optical flow of two adjacent frames. The opposite direction of the optical flow is used to identify the corresponding position in the previous frame. The coordinates of the corresponding positions in the preceding and following frames is defined as $L_{former}=\left(x-\Delta x,y-\Delta y\right)$ and $L_{later}=\left(x+\Delta x,y+\Delta y\right)$. We use tri-linear interpolation from the eight corner points of the voxel to calculate the output value P(x,y) by four points: ${\overset{ˇ}{X}}_{00}\left({\overset{ˇ}{x}}_{ceil},{\overset{ˇ}{y}}_{ceil}\right)$, ${\overset{ˇ}{X}}_{01}\left({\overset{ˇ}{x}}_{ceil},{\overset{ˇ}{y}}_{floor}\right)$, ${\overset{ˇ}{X}}_{10}\left({\overset{ˇ}{x}}_{floor},{\overset{ˇ}{y}}_{ceil}\right)$, and ${\overset{ˇ}{X}}_{11}\left({\overset{ˇ}{x}}_{floor},{\overset{ˇ}{y}}_{floor}\right)$, from the former frame, and the other four points: ${\hat{X}}_{00}\left({\hat{x}}_{ceil},{\hat{y}}_{ceil}\right)$, ${\hat{X}}_{01}\left({\hat{x}}_{ceil},{\hat{y}}_{floor}\right)$, ${\hat{X}}_{10}\left({\hat{x}}_{floor},{\hat{y}}_{ceil}\right)$ and ${\hat{X}}_{11}\left({\hat{x}}_{floor},{\hat{y}}_{floor}\right)$, from the later frame.

The time component of the voxel stream *F* can be considered as a linear blending weight between two adjacent frames. We employ this voxel stream to sample the input two frames and use the volume sampling function $T_{x,y,\omega }$ to generate the final predicted frame *P*.

(9) $$\begin{array}{cc} P_{inter}\left(x,y\right) & =T_{x,y,\omega }\left(X_{rec},H\left(X_{rec};\Theta \right)\right)\\ \; & =\omega \cdot P_{former}\left(x,y\right)+\left(1-\omega \right)\cdot P_{later}\left(x,y\right), \end{array}$$

where $P_{former}\left(x,y\right)$ and $P_{later}\left(x,y\right)$ are computed by

(10) $$\begin{array}{c} P_{former}\left(x,y\right)=\left[\begin{array}{cc} 1-x & x \end{array}\right]\times \left[\begin{array}{cc} {\overset{ˇ}{X}}_{00} & {\overset{ˇ}{X}}_{01}\\ {\overset{ˇ}{X}}_{10} & {\overset{ˇ}{X}}_{11} \end{array}\right]\times \left[\begin{array}{c} 1-y\\ y \end{array}\right],\\ P_{later}\left(x,y\right)=\left[\begin{array}{cc} 1-x & x \end{array}\right]\times \left[\begin{array}{cc} {\hat{X}}_{00} & {\hat{X}}_{01}\\ {\hat{X}}_{10} & {\hat{X}}_{11} \end{array}\right]\times \left[\begin{array}{c} 1-y\\ y \end{array}\right]. \end{array}$$

The interpolation network adopts a fully convolutional structure, with four convolutional layers and four deconvolutional layers. To better maintain the spatial features in the low dimension layer, some jump connections between the corresponding convolutional layer and deconvolutional layer are added.

### 5.3. Inter Loss Function Design

The prediction module is represented by $H(X_{\left(t0,t0+2k\right)},\Theta )$, where the output $P_{t0+k}$ is the predicted point cloud at t=T+1 and Θ is the network parameters.

(11) $$P_{t0+k}=H\left(X_{\left(t0,t0+2k\right)},\Theta \right),$$

The predited point cloud data are converted to range image, and the difference between the original range image $X_{t0+k}\left(x,y\right)$ and the predicted range image $P_{t0+k}\left(x,y\right)$ is calculated as the residual data $R_{inter}\left(x,y\right)$, which will be encoded losslessly. Explicity, the training loss is defined as follows:

(12) $$\begin{array}{cc} R_{inter}\left(x,y\right) & =X_{t0+k}\left(x,y\right)-P_{t0+k}\left(x,y\right)\\ \; & =X_{t0+k}\left(x,y\right)-H\left(X_{\left(t0,t0+2k\right)},\Theta \right) \end{array}$$

(13) $$L_{loss}=\left|\right|R_{inter}{\left|\right|}_{2}$$

### 5.4. Visualization Results

**Figure 4:**
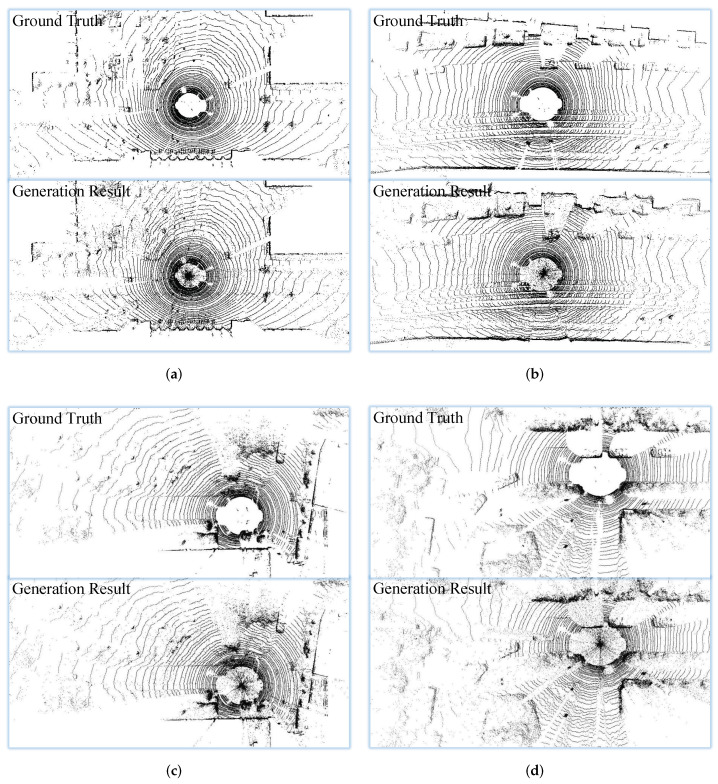
Qualitative results: (**a**) campus; (**b**) city; (**c**) road; (**d**) residential (Best viewed by zooming in).

For the inter-coding network, if the point-cloud interpolation module synthesizes more accurate point clouds, smaller residual data and higher compression performance can be achieved. To deeply evaluate, four scenes point cloud interpolation results are obtained, as illustrated in Figure 4. We can see that the predicted point cloud and the original point cloud are almost the same. The effectiveness of the inter insertion module is confirmed.

## 6. Experimental Results

The proposed point cloud compression scheme is implemented in python using point cloud libraries (PCL) [35] on a PC with a TITAN RTX GPU. The KITTI dataset [36], including city, residential, campus, and road scenes, is used to evaluate our algorithm.

### 6.1. Evaluation Metric

To evaluate the overall performance, the calculation of the compression ratio (CR) and relative distance ($D_{d}$) is considered. The CR is obtained by calculating the ratio between the point cloud size after compression and the original size.

(14) $$CR=\frac{Compressed_{size}}{Original_{size}}\times 100\%,$$

$D_{d}$ represents the distance between the ground truth LiDAR data $P_{input}$ and the reconstructed one $P_{decode}$. $D_{d}$ is defined as follows:

(15) $$\begin{array}{cc} D_{d}\left(P_{input},P_{decode}\right) & =\frac{{\bar{D}}_{d}\left(P_{input},P_{decode}\right)}{2}+\frac{{\bar{D}}_{d}\left(P_{decode},P_{input}\right)}{2}\\ {\bar{D}}_{d}\left(P_{i},P_{j}\right) & =\frac{1}{|P_{i}|}\sum_{x_{i}\in P_{i}}\min_{x_{j}\in P_{j}}d\left(x_{i}-x_{j}\right) \end{array}.$$

The measure is sensitive to false positives (rebuilding points in unoccupied areas) and false negatives (excluding occupied areas).

### 6.2. Coding Performance for a Single Frame

**Figure 5:**
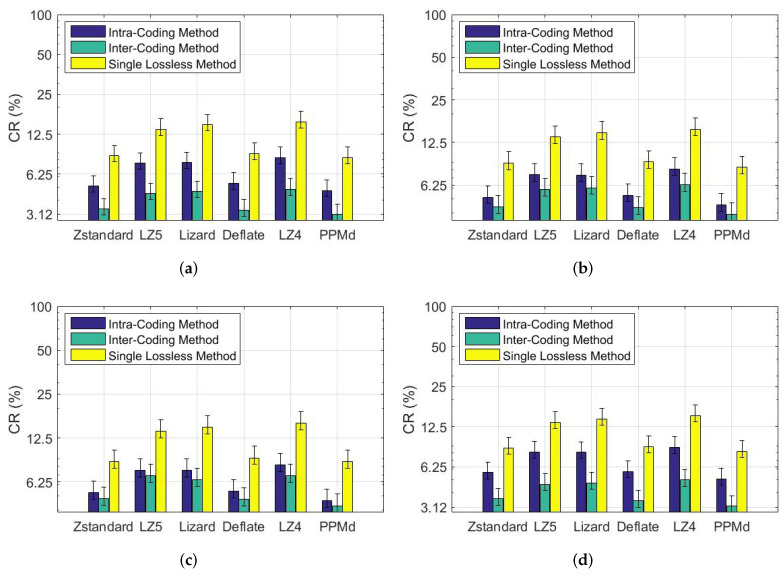
CR of different lossless coding schemes: (**a**) campus; (**b**) city; (**c**) road; (**d**) residential.

To find the most efficient coding method for the residual data, several lossless coding schemes are used to encode the residual data, including *Zstandard*, *LZ5*, *Deflate*, *Lizard*, *LZ4*, and *PPMd*. To verify the compression efficiency in removing the time and space redundancy, the 2D matrices are also directly encoded with the lossless coding algorithms without any preprocessing. Figure 5 shows the experimental results.

For intra-coding, the smallest CR value is 4.64%, achieved by using the *PPMd* scheme for the *city* scene due to its simple structure. The point cloud of the residential scene, however, is complex and the CR is much higher. The smaller the CR, the better the coding performance.

For inter-coding, it can be observed that using *the PPMd* scheme achieves the best compression performance for the *campus* scene, with a CR of 3.09%. The worst CR is 6.87% for the *residential* scene using the *LZ4* scheme. However, the CR of the proposed inter-coding network is still smaller than that of directly coding point cloud data with lossless coding schemes.

### 6.3. Comparsion with Octree and Draco

According to the time stamp and quaternion matrix of each scan, the scans are merged into a LiDAR panoramic map. Taking this point cloud map as a whole, Table 1 describes the CR results of the proposed coding method compared to *Octree* [35] and Google Draco [28]. The quantization accuracy (QA) of our method is set to 2 mm, 5 mm, and 1 cm, while the distance resolution (DR) of *Octree* is set to 1 mm${}^{3}$, 5 mm${}^{3}$, and 1 cm${}^{3}$. The quantization bits (QB) of Draco are set to 17, 15, and 14, which correspond to 1 mm accuracy, 5 mm accuracy, and 1 cm accuracy, respectively. Besides that, the compression level (CL) = 10 is set to achieve the highest compression rate. In our experiments, *PPMd* is selected to encode residual data. From Table 1, it can be observed that compared with *Octree*, the proposed algorithm achieves a smaller CR value.

### 6.4. Rate-Distortion Curves

**Figure 6:**
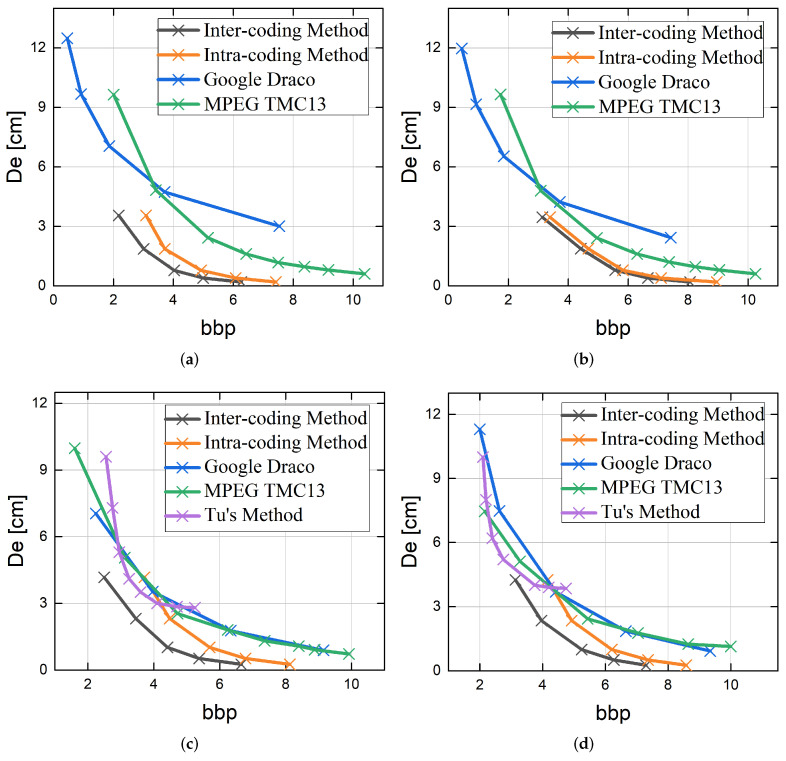
$D_{d}$-bpp curves of our method in compared to Google Draco [28], MPEG TMC13 [20] and Tu’s method [26]: (**a**) *campus*, (**b**) *city*, (**c**) *road*, and (**d**) *residential*.

Four state-of-the-art baselines are chosen for comparison, including Google Draco [28], MPEG TMC13 [20], and Tu’s method [26]. The results are evaluated in terms of the relationship between the Distance and the bit per point (bpp) for point cloud data for four scenes, as illustrated in Figure 6. The proposed LiDAR point clouds coding method has shown outstanding advantages in terms of bbp and $D_{d}$ among these methods [37,38,39]. Google Draco and MPEG TMC13 are not designed specifically for multi-line LiDAR data [40,41]. Compared to these methods, our method can generate the point cloud more accurately, which contributes to largely removing the redundancy and obtaining a better $D_{d}$-bpp result.

### 6.5. Computational Complexity

The proposed intra-coding consists of three steps, namely segmentation, intra-prediction, and residual data coding, while the inter-coding method consists of inserting frame and residual data coding. By calculating the average coding time, 100 frames are selected. Experimental results show that the total intra-coding time with the lossless method (PPMd) is 0.21 s and the total inter-coding time is 0.15 s.

## 7. Discussion

With a 5–15 HZ user-selectable frame rate, the LiDAR sensor of HDL-64E S2 captures over 1.3 million points per second. The drawback of the proposed LiDAR point cloud map coding scheme is that the intra-coding and inter-coding can not meet the real-time requirement [42,43,44]. However, it can be used for off-line LiDAR point cloud map coding to reduce its storage space and transmission bandwidth, which can be used in mobile robots or surveying and mapping fields. The follow-up research focuses on implementing the coding scheme to the FPGA platform to accelerate the algorithm and achieve real-time performance.

## 8. Conclusions

Ranging sensors, such as LiDAR, are considered to be very robust under all light conditions or foggy weather, which have been widely used in the field of autonomous driving tasks, for instance, navigation, obstacle avoidance, target tracking, and recognition, etc. However, the enormous volume of LiDAR point clouds brings great challenges to data storage and transmission. To address this issue, this paper focuses on LiDAR point cloud map coding. Firstly, an intra-coding technique is designed based on point cloud segmentation and geometric reconstruction, which can effectively remove the spatial redundancy of the LiDAR point cloud. Secondly, we designed a point cloud insertion network to remove the time redundancy of point clouds by inserting frames into the intermediate moment according to the encoded point clouds. Experiments demonstrate that the proposed method obtains a higher comparison performance compared with several representative point cloud methods.

## Figures and Tables

**Table 1:** Comparison ratio results with the *Draco* and *Octree* methods.

	Inter-Inserting Method			*Octree* [35]			*Draco* [28]		
**Scene**	**QA**			**DR**			**QB**		
	**2 mm**	**5 mm**	**1 cm**	**1 mm^3^**	**5 mm^3^**	**1 cm^3^**	**17 (bits)**	**15 (bits)**	**14 (bits)**
Campus	3.09	2.49	2.01	21.27	8.05	5.75	11.87	7.75	5.47
City	3.90	3.38	2.83	23.98	10.76	8.40	12.52	8.38	6.49
Road	3.16	0.26	2.12	23.56	10.35	7.99	12.31	8.35	6.59
Residential	4.29	3.68	3.16	22.94	9.72	7.37	12.66	8.59	6.33
Average	3.61	3.03	2.53	20.23	9.72	7.29	12.34	8.27	6.22

## Data Availability

Not applicable.
